# Elimination of Porcine Epidemic Diarrhea Virus in an Animal Feed Manufacturing Facility

**DOI:** 10.1371/journal.pone.0169612

**Published:** 2017-01-18

**Authors:** Anne R. Huss, Loni L. Schumacher, Roger A. Cochrane, Elizabeth Poulsen, Jianfa Bai, Jason C. Woodworth, Steve S. Dritz, Charles R. Stark, Cassandra K. Jones

**Affiliations:** 1 Department of Grain Science and Industry, Kansas State University, Manhattan, Kansas, United States of America; 2 Department of Diagnostic Medicine/Pathobiology, Kansas State University, Manhattan, Kansas, United States of America; 3 Department of Animal Sciences and Industry, Kansas State University, Manhattan, Kansas, United States of America; University of Kansas Medical Center, UNITED STATES

## Abstract

Porcine Epidemic Diarrhea Virus (PEDV) was the first virus of wide scale concern to be linked to possible transmission by livestock feed or ingredients. Measures to exclude pathogens, prevent cross-contamination, and actively reduce the pathogenic load of feed and ingredients are being developed. However, research thus far has focused on the role of chemicals or thermal treatment to reduce the RNA in the actual feedstuffs, and has not addressed potential residual contamination within the manufacturing facility that may lead to continuous contamination of finished feeds. The purpose of this experiment was to evaluate the use of a standardized protocol to sanitize an animal feed manufacturing facility contaminated with PEDV. Environmental swabs were collected throughout the facility during the manufacturing of a swine diet inoculated with PEDV. To monitor facility contamination of the virus, swabs were collected at: 1) baseline prior to inoculation, 2) after production of the inoculated feed, 3) after application of a quaternary ammonium-glutaraldehyde blend cleaner, 4) after application of a sodium hypochlorite sanitizing solution, and 5) after facility heat-up to 60°C for 48 hours. Decontamination step, surface, type, zone and their interactions were all found to impact the quantity of detectable PEDV RNA (*P* < 0.05). As expected, all samples collected from equipment surfaces contained PEDV RNA after production of the contaminated feed. Additionally, the majority of samples collected from non-direct feed contact surfaces were also positive for PEDV RNA after the production of the contaminated feed, emphasizing the potential role dust plays in cross-contamination of pathogen throughout a manufacturing facility. Application of the cleaner, sanitizer, and heat were effective at reducing PEDV genomic material (*P* < 0.05), but did not completely eliminate it.

## Introduction

The United States swine industry has suffered dramatic losses following the emergence of porcine epidemic diarrhea virus (PEDV) in May 2013. The virus is a highly contagious and deadly coronavirus that was only observed in Asian and European swine herds prior to 2013. Due to the high mortality rates (80–100%) in neonates, significant economic losses have been experienced [1, 2]. Traditionally, transmission of PEDV occurs through the fecal-oral route within a herd with acutely infected pigs shedding large quantities of the virus for several days after infection. Due to the large amount of virus that can be shed by infected herds, there is potential for the virus to contaminate facility surroundings, leading to contamination issues off-property. Other modes of transmission, in addition to infected pigs, include fecal contamination of animal transport vehicles, PEDV-positive aerosols, and contaminated animal feed or ingredients [1, 3, 4, 5, 6]. Observations also indicate presence of PEDV genetic material in feed transport vehicles suggesting these vehicles may be a potential vector [7].

With the confirmation of feed manufacturing related vectors for PEDV transmission, the potential for contamination of feed manufacturing facilities with PEDV exists. Traditionally, feed manufacturing facilities rely on good manufacturing practices, physical cleaning, removal of feedstuff residues and dust, employee hygiene and effective pest management to control biological hazards [8, 9, 10, 11]. However, these protocols have been developed based on eliminating *Salmonella* or other bacteria with no information documenting the elimination of viral contamination from feed production facilities.

Previous research has indicated that a decontamination protocol of physical cleaning with liquid detergents and sanitizers and heat was effective at elimination of *Enterococcus faecium*, a surrogate for *Salmonella*, in a feed manufacturing facility [10]. Therefore, the objective of this study was to evaluate this standard decontamination protocol for elimination of PEDV from a feed manufacturing facility. Efficacy of the standardized decontamination protocol was monitored by collection of environmental samples (swabs) during the manufacture of mash diet inoculated with PEDV subsequent cleanup following the established decontamination protocol.

## Materials and Methods

For evaluation of the decontamination protocol to reduce or eliminate PEDV from feed processing equipment and processing facilities, the Cargill Feed Safety Research Center (FSRC) in the O.H. Kruse Feed Technology Innovation Center at Kansas State University was used. The FSRC is a biosafety level-2 (BSL-2) laboratory with pilot scale feed manufacturing equipment. The FSRC includes a containment mode, where a separate air handling system with HEPA filtration and biosafety protocols restrict pathogen exit during operation of equipment. Employees are required to wear personal protective equipment and follow strict biosafety guidelines while operating in containment mode. As a final decontamination step, the entire temperature of the facility can be raised to and maintained at 60°C. Due to the dual functionality of the FSRC as a pilot-scale feed mill operated not under containment, effective decontamination is essential to prevent the contamination of research feeds. Thawing of the virus, preparation of the inoculum and subsequent inoculation of the larger feed batch was all done within the FSRC in containment mode. The FSRC was not removed from containment mode until completion of decontamination after the final replication.

### Environmental Sample Collection and Analysis

All environmental samples were collected using swabs (World Bio-Products LLC, Woodinville, WA) pre-wetted with sterile 1X phosphate buffered saline pH 7.4 (Life Technologies, Grand Island, NY) by rubbing the moist swab across the desired surface. Swab samples were collected from designated locations within the FSRC at baseline (prior to the manufacture of contaminated feed), after production of PEDV-contaminated feed and after each step of the decontamination protocol [10]. All collected swabs were analyzed for PEDV RNA by reverse transcriptase-quantitative polymerase chain reaction (RT-qPCR).

For RT-qPCR analysis, the swab tubes were vortexed briefly before 50 μL of fluid was transferred to a 96-well plate and used for RNA extraction. Automated extraction was carried out on a KingFisher magnetic particle processor (Thermo Scientific, Waltham, MA) using a MagMAX™-96 Viral RNA Isolation Kit (Life Technologies, Grand Island, NY). All manufacturer’s instructions were followed, with the exception of a final elution volume of 60 μl. Each 96-well extraction plate included an extraction positive control (PEDV inoculum) and an extraction negative control (1x PBS).

A duplex real-time RT-PCR (RT-qPCR) targeting the Spike gene of PEDV and host 18S rRNA (internal control) was used for the detection and quantification of PEDV. Primer and probe information is listed in Table 1. The RT-qPCR was carried out in 20 μl reaction volumes with final concentrations of: 1x Path-ID™ Multiplex RT-PCR buffer, 2 μl Path-ID™ Multiplex Enzyme Mix (Applied Biosystems, Foster City, CA), 500 nM of each primer, 62.5 nM of each probe, and 4 μl sample RNA. The reactions were carried out on the CFX96 Touch™ Real-Time PCR Detection System (Bio-Rad Laboratories, Hercules, CA) and consisted of 10 min reverse transcription at 48°C, 10 min of reverse transcriptase inactivation/initial denaturation at 95°C followed by 45 cycles of 10 s at 95°C and 40 s at 60°C. Each 96-well RT-qPCR plate included the two extraction controls (above) and a PCR positive control (known PEDV-positive RNA sample) and a PCR negative control (water). Results were analyzed using the CFX Manager™ Software (Bio-Rad Laboratories, Hercules, CA). 18S Ct values were monitored for each sample as an internal positive extraction control.

**Table 1:** Primer and probe sequences used in the duplex real-time RT-PCR (RT-qPCR) assay for the detection and quantification of environmental swabs for PEDV.

Primer/ Probe	ID	Sequence (5’—3’)	Fluorescent Dye	Quencher
PEDV Probe	wPr3a	AAAGGCTCTTGCGAAATGCC	FAM	BHQ-1
PEDV Forward Primer	wF3a	TGCTAGTGGCGTTCATGGTAT		
PEDV Forward Primer	wF3b	ACTGATAGTGGCGTTCATGGTAT		
PEDV Reverse Primer	R3	TGTAAATAAAGCTGGTAACCACTAGG		
18S Probe	18S-Pr	AAGGAATTGACGGAAGGGCA	Cy5	BHQ-2
18S Forward Primer	18S-F	GGAGTATGGTTGCAAAGCTGA		
18S Reverse Primer	18S-R	GGTGAGGTTTCCCGTGTTG		

### Preparation of Inoculum

U.S. PEDV prototype strain cell culture isolate USA/IN/2013/19338, passage 8 (PEDV19338) that contained 4.5 × 10^6^ tissue culture infectious dose (TCID_50_/ml) was used to inoculate the feed. This same PEDV from the same passage had been demonstrated as pathogenic using bioassay [12]. The virus was divided into three, 500 ml aliquots with one aliquot used in each replication. At the beginning of each replication, one 500 mL aliquot was thawed overnight in the refrigerator and used to prepare the feed inoculum. To prepare the inoculum, 500 mL of the thawed virus was mixed with 4.5 kg of the corn-soybean meal-based diet.

### Inoculation of Diet and Feed Production

The previously prepared inoculum was added to 45 kg of a corn-soybean meal-based diet in a 0.113 m^3^ electric paddle mixer (H.C. Davis Sons Manufacturing, Bonner Springs, KS) and mixed for 5 minutes. The mixed feed was then discharged at a rate of approximately 4.5 kg per minute into the leg of the bucket elevator (Universal Industries, Cedar Falls, IA). The feed exited the bucket elevator through a downspout, collected, and then converted to pellets using a pilot-scale single pass conditioner and pellet mill (Model CL5, CPM, Waterloo, IA).

### Facility Decontamination Protocol

Upon completion of converting the inoculated mash into pellets, remaining organic material on all equipment and surfaces was physically removed followed by application of a quaternary ammonium-glutaraldehyde blend cleaner (Alkyl (C12 67%, C14 25%, C16 7%, C18 1%) dimethyl benzyl ammonium chloride 26%, glutaraldehyde 7% and inert ingredients 67%; Synergize^TM^, diluted 1:256, Preserve International, Reno, NV). This was followed by sanitizing with a 5% sodium hypochlorite solution (5% solution in water; Chlorox, Oakland, CA) in water, applied to all equipment and facility surfaces. Both disinfectants were applied using a pressure washer (LANDA, Camas, WA; 2200 PSI; 80°C) in a top to bottom fashion with all equipment surfaces, walls and floors thoroughly saturated. Once all chemical disinfection was completed, the entire FSRC facility was heated via an internal system, which monitored air temperature on each floor of the facility. During facility heat-up, a temperature of 60°C or higher was maintained for a minimum of 48 hours. Upon completion of 48 hours at 60°C, the heat-up system was turned off and the facility was allowed to cool back to ambient temperature.

### Statistical Analysis

The goal of the decontamination protocol was to completely eliminate PEDV at a detectable level when measured by RT-qPCR. When evaluating RT-qPCR result, Ct values were translated to positive/negative for statistical evaluation with a Ct of ≥ 40 considered negative. Results were analyzed using the proc GLM procedure of SAS using the fixed effects of decontamination step, surface (concrete, metal, plastic or rubber), type (structural vs equipment), and zone (1, 2, or 3). All interactions were considered, but non-significant interactions were removed from the model statement. Results were considered significant at *P* < 0.05.

## Results and Discussion

Porcine epidemic diarrhea virus (PEDV) has had devastating impacts to the swine industry as it spread throughout the U.S. Identification of feed related vectors for transmission has led to increased research to identify methods of PEDV-mitigation in finished feeds and feed ingredients. However, monitoring and elimination of PEDV within animal feed manufacturing facilities has received little attention from researchers. To our knowledge, this is the first study to evaluate the presence of PEDV within an animal feed manufacturing facility during the manufacture of a swine diet artificially inoculated with PEDV and the subsequent decontamination of the facility.

Prior to the beginning of each replicate, subsamples of the feed to be used were evaluated for PEDV RNA. As expected all feed was negative, with no detectable PEDV RNA found via RT-qPCR analysis. In addition, all baseline swabs collected during each replication were negative for PEDV. After the introduction of PEDV-inoculum to the feed in each replicate run, three subsamples of inoculated feed were collected and evaluated for PEDV RNA by RT-qPCR. The PEDV cell culture had Ct values of 16.7, 15.9, and 16.2 for replicates 1, 2, and 3, respectively. While the PEDV-inoculated feed contained average Ct values of 32.6, 31.4, and 32.2 for replicates 1, 2, and 3, respectively. This is consistent with previously observed PEDV contaminated feed [13]. Infectivity of the feed was further confirmed by bioassay in 10 d old pigs.

As previously mentioned, all collected environmental swabs were categorized by decontamination step, surface collected from (concrete, metal, plastic, or rubber), surface type (equipment or structural) and zone (1, 2, or 3), with results reported as number of positive samples out of total samples collected (Table 2). As previously described by Huss et al. [10], zones were categorized based on proximity to feed contact surfaces, with zone 1 including direct feed contact surfaces; zone 2 included surfaces immediately adjacent to direct contact surfaces (exteriors of equipment, conveyor housing, etc.); zone 3 included non-feed contact surfaces outside of zone 1 and 2 but within the FSRC (floors, walls, drains, etc.). The main effects of decontamination step, surface (concrete, metal, plastic, or rubber), type (equipment or structural), and zone (1, 2, or 3) are shown in Table 3. All main effects and interactions were found to be significant (*P* < 0.05).

**Table 2:** Number of swabs with detectable PEDV RNA compared to the number of swabs collected, based on surface characteristic and type, collection zone and decontamination step done immediately prior to sample collection.

Sample Location	Baseline^a^	After production of inoculated feed^a^	After chemical cleaning with ammonium-glutaraldehyde blend^a^	After chemical cleaning with sodium hypochlorite^a^	After facility heat-up to 60°C for 48 hours^a^
Surface					
Concrete	0/15	6/6	1/8	1/8	0/13
Metal	0/33	31/33	8/33	5/33	2/31
Plastic	0/3	12/12	1/3	0/3	0/4
Rubber	0/6	15/15	2/6	0/6	0/5
Type					
Equipment	0/33	51/51	10/33	4/33	2/31
Structural	0/24	13/15	2/17	0/17	0/21
Zone					
1	0/27	36/36	8/27	4/27	2/27
2	0/3	12/12	2/3	0/3	0/2
3	0/27	16/18	2/20	0/20	0/23

^a^Values are number of positive samples/number of samples tested; positive considered a Ct value of ≤ 40.

**Table 3:** Main effects and interactions of factors considered for building decontamination.

Factor	*P* =
Main effects	
Decontamination step^a^	< 0.0001
Surface^b^	0.0018
Type^c^	0.0010
Zone^d^	< 0.0001
Two-way interactions	
Decontamination step × surface	< 0.0001
Decontamination step × type	< 0.0001
Decontamination step × zone	< 0.0001
Surface × type	0.0002
Surface × zone	0.0002
Type × zone	0.0002
Three-way interactions	
Decontamination step × surface × type	< 0.0001
Decontamination step × surface × zone	< 0.0001
Decontamination step × type × zone	< 0.0001
Surface × type × zone	0.0002
Four-way interactions	
Decontamination step × surface × type × zone	< 0.0001

^a^Compared sampled collected at each of the 5 decontamination steps, including: 1) baseline measurements prior to inoculation, 2) immediately after feed manufacturing production, 3) chemical cleaning using a quaternary ammonium-glutaraldehyde blend, 4) chemical cleaning using sodium hypochlorite, and 5) facility heat-up to 60°C for 48 hours.

^b^Compared samples collected on concrete, metal, plastic, or rubber.

^c^Compared samples collected on surfaces that were from equipment or structural.

^d^Compared samples collected on each of zones 1, 2, and 3.

**Fig 1:**
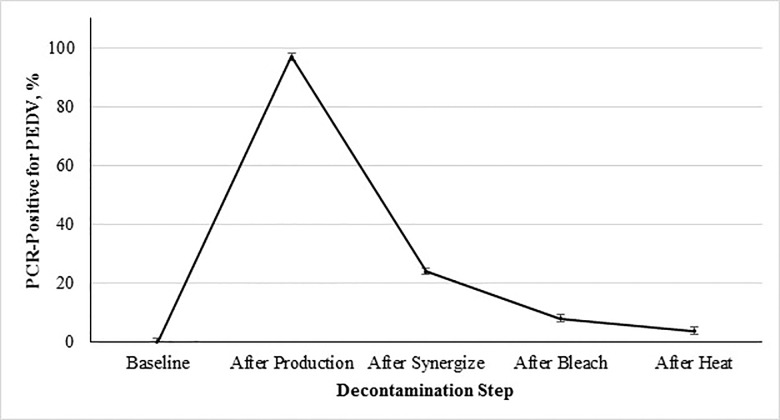
Percent PCR-positive for PEDV from environmental samples collected during decontamination as a function of step of the decontamination protocol followed after processing PEDV-inoculated mash feed into pellets.

The percent contamination of PEDV after each step of the decontamination protocol are shown in Fig 1, and illustrates the increase in PEDV genetic material found throughout the facility after production of contaminated feed. All baseline samples collected contained no detectable PEDV, which increased to 192 of 198 (97.0%) samples positive for PEDV RNA after production of the contaminated feed (Fig 1). Following cleaning with the ammonium-glutaraldehyde blend 36 of 150 (24.0%) of the collected samples were positive for PEDV RNA (Fig 1). This was further reduced to 14 of 150 collected samples positive after sanitization with the sodium hypochlorite solution (Fig 1). Following facility heat-up to 60°C for 48 hours, 6 of 157 (3.8%) of the collected samples were positive for PEDV RNA (Fig 1). It is important to note that all 6 of the positive samples found after heat-up were from replication 2. Due to these results, the decontamination process was repeated (cleaning with the ammonium-glutaraldehyde blend, followed by sanitization with a sodium hypochlorite solution and facility heat-up to 60°C for 48 hours) and the collection of additional swabs. All swabs collected after the repeat decontamination were negative for PEDV RNA. Overall the efficacy of the chemical disinfection and heat to decontaminate the facility were consistent with previous research done for decontamination of animal transport vehicles [14].

Main effect of decontamination step on incidence of sample contamination with PEDV (*P* < 0.0001; SEM = ±1.21). The 5 evaluated decontamination steps were: 1) baseline prior to inoculation, 2) after feed production, 3) chemical cleaning with a quaternary ammonium-glutaraldehyde blend, 4) chemical cleaning with sodium hypochlorite, and 5) facility heat-up to 60°C 48 hours. Note: Positive swabs were obtained after decontamination during replication 2, therefore decontamination was repeated with subsequent swabs collected and found negative.

**Fig 2:**
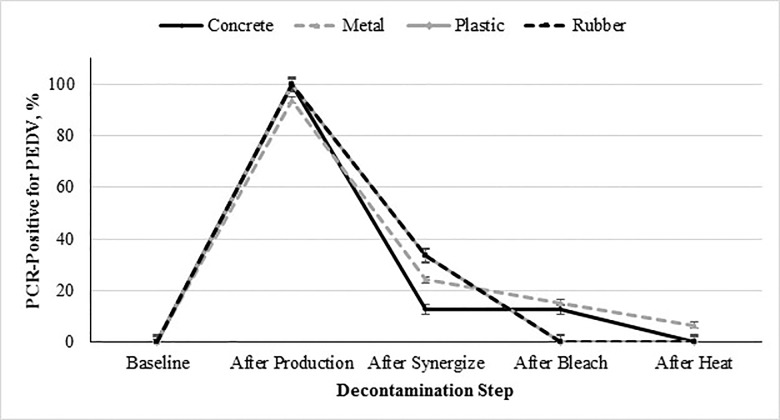
Percent PCR-positive for PEDV from environmental samples collected based on surface composition (concrete, metal, plastic or rubber) during decontamination as a function of step of the decontamination protocol followed after processing PEDV-inoculated mash feed into pellets.

The percent contamination of PEDV on each surface type (concrete, metal, plastic, or rubber) from baseline to the final decontamination step is shown in Fig 2. As stated previously, the surface main effect was significant (*P* = 0.0018; Table 3). Plastic and rubber surfaces were not significantly different from each other, but were both significantly different from concrete and metal surfaces. Additionally, concrete and metal surfaces were not significantly different from each other. The differences between the plastic and rubber vs concrete and metal surfaces could be attributed the specific characteristics of each surface type. Specifically, the plastic and rubber surfaces were smoother compared to the concrete and metal surfaces. Both the concrete and metal surfaces were rough, with concrete also being porous and metal being pitted from use. The smooth attributes of the plastic and rubber surfaces are conducive to decontamination.

Interactive effect of decontamination step × surface (concrete, metal, plastic, and rubber) on incidence of sample contamination with PEDV (*P <* 0.0018; SEM for concrete, metal, plastic and rubber = ±2.05, ±1.14, ±2.91, and ±2.39, respectively). The 5 evaluated decontamination steps were: 1) baseline prior to inoculation, 2) after feed production, 3) chemical cleaning with a quaternary ammonium-glutaraldehyde blend, 4) chemical cleaning with sodium hypochlorite, and 5) facility heat-up to 60°C 48 hours. Note: Positive swabs were obtained after decontamination during replication 2, therefore decontamination was repeated with subsequent swabs collected and found negative.

**Fig 3:**
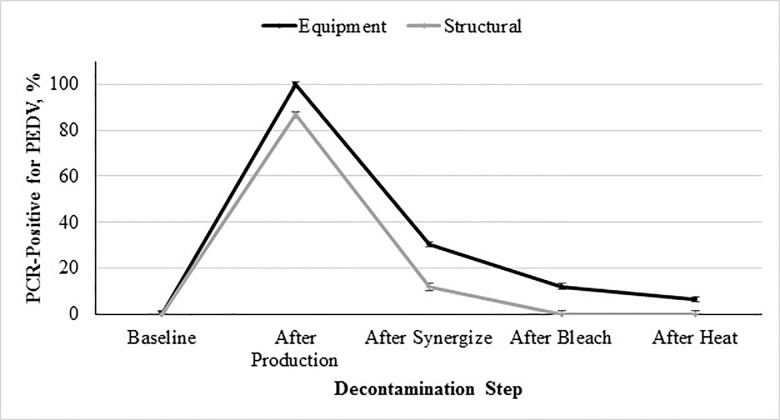
Percent PCR-positive for PEDV from environmental samples collected based on surface type (equipment or structural) during decontamination as a function of step of the decontamination protocol followed after processing PEDV-inoculated mash feed into pellets.

The same trend of high contamination after production and subsequent reduction during decontamination was observed in samples collected from the two different surface types (equipment or structural), as shown in Fig 3. All swabs collected from equipment surfaces contained PEDV RNA after processing of the contaminated feed. The quantity of viral RNA between the equipment and structural swabs was statistically different, with greater contamination found in equipment swabs (*P* < 0.05). This was expected due to the equipment being in direct contact with the contaminated feed. The majority of collected structural swabs were also positive, but remained less than those collected from equipment throughout the experiment. This was also as expected as structural swabs did not have direct contact with the contaminated feed. It is important to note the extent of contamination of structural surfaces, thus emphasizing the important role dust particles may play in the spread of biological hazards throughout a feed manufacturing facility. These results are congruent with previous research implementing dust in the spread of bacterial biological hazards within a feed manufacturing facility [8, 10]. To our knowledge this is the first report to describe the spread of a viral pathogen through the environment of a feed production facility.

Interactive effect of decontamination step × type (equipment or structural) on incidence of sample contamination with PEDV (*P* < 0.001; SEM for equipment and structural = ±1.12 and ±1.50, respectively). The 5 evaluated decontamination steps were: 1) baseline prior to inoculation, 2) after feed production, 3) chemical cleaning with a quaternary ammonium-glutaraldehyde blend, 4) chemical cleaning with sodium hypochlorite, and 5) facility heat-up to 60°C 48 hours. Note: Positive swabs were obtained after decontamination during replication 2, therefore decontamination was repeated with subsequent swabs collected and found negative.

**Fig 4:**
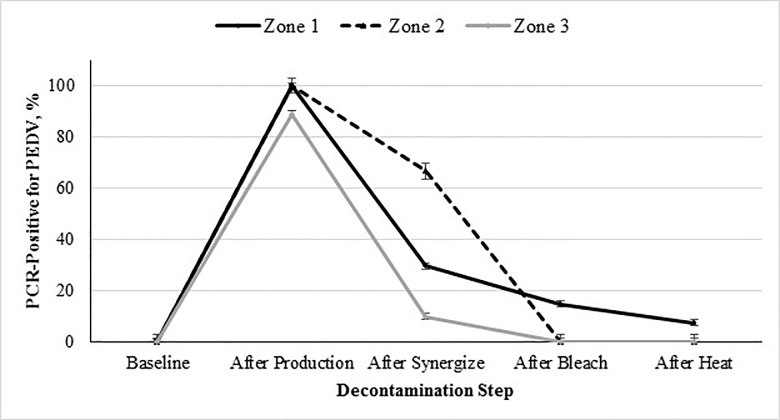
Percent PCR-positive for PEDV from environmental samples collected from zones 1, 2, and 3 during decontamination as a function of step of the decontamination protocol followed after processing PEDV-inoculated mash feed into pellets.

Again, the same overall trend was observed when looking at swabs collected from the different zones (1, 2, or 3), as shown in Fig 4. Zones 1 and 2 had the highest amount of contamination compared to zone 3, as they were in direct or adjacent contact with the contaminated feed. Significantly less contamination was seen in zone 3 (vs zones 1 and 2; *P* < 0.05), as expected due to the proximity to the contaminated feed. However, as seen with the facility swabs, dust generated during processing of contaminated feed can lead to contamination of previously uncontaminated surfaces. This is consistent with data indicating that aerosolized dust can be a transmission vector [15]. This previous work indicates that the PEDV particles can be attached to dust. Based on our results this suggests that contaminated dust may be circulated throughout the manufacturing facility.

Interactive effect of decontamination step × zone (1, 2, and 3) on incidence of sample contamination with PEDV (*P* < 0.0001; SEM for zone 1, 2 and 3 = ±1.20, ±2.99, and ±1.38, respectively). The 5 evaluated decontamination steps were: 1) baseline prior to inoculation, 2) after feed production, 3) chemical cleaning with a quaternary ammonium-glutaraldehyde blend, 4) chemical cleaning with sodium hypochlorite, and 5) facility heat-up to 60°C 48 hours. Note: Positive swabs were obtained after decontamination during replication 2, therefore decontamination was repeated with subsequent swabs collected and found negative.

These findings are important to illustrate that wet chemical cleaning greatly reduced the sample positive rates then followed by facility heating were effective measures to reduce PEDV in feed manufacturing facilities and equipment. These are consistent with protocols to document disinfection of pig transport vehicles [14]. Although effective, the wet chemical cleaning and facility heating are impractical on a large-scale basis for feed manufacturing facilities. These findings may provide solutions for biological hazard reduction on specific equipment or problem areas. It is also important to note the importance of equipment cleaning, as positive swabs were found after the final decontamination step (during replication 2, as stated previously).

## Conclusions

In conclusion, this experiment demonstrated the magnitude of cross-contamination possible when PEDV enters a feed manufacturing facility. The virus was spread to nearly all feed- and non-feed-contact surfaces and was particularly challenging to decontaminate, even with the aid of wet sanitizers and heat. Therefore, feed mill operators must be particularly judicious regarding use of good manufacturing practices and biosecurity protocols to exclude the virus from entry into the feed mill. Furthermore, additional research is necessary to understand the role of dust in animal food biological hazard transmission and to formulate practical feed mill sanitation recommendations to prevent and reduce PEDV contamination of equipment and facilities.
